# A comprehensive deep learning approach to improve enchondroma detection on X-ray images

**DOI:** 10.1038/s41598-025-07978-4

**Published:** 2025-08-20

**Authors:** Ayhan Aydin, Caner Ozcan, Safak Aydın Simsek, Ferhat Say

**Affiliations:** 1 Faculty of Engineering, Ondokuz Mayis University, 55200 Atakum, Samsun, Turkey; 2 Faculty of Engineering, Karabuk University, 78050 Kılavuzlar, Karabuk, Turkey; 3 Faculty of Medicine, Ondokuz Mayıs University, 55200 Atakum, Samsun, Turkey

**Keywords:** Enchondroma, Deep learning, Yolo, Detectron, Radiograph, Tumor, Machine learning, Bone cancer, Preventive medicine, Electrical and electronic engineering

## Abstract

An enchondroma is a benign neoplasm of mature hyaline cartilage that proliferates from the medullary cavity toward the cortical bone. This results in the formation of a significant endogenous mass within the medullary cavity. Although enchondromas are predominantly asymptomatic, they may exhibit various clinical manifestations contingent on the size of the lesion, its localization, and the characteristics observed on radiological imaging. This study aimed to identify and present cases of bone tissue enchondromas to field specialists as preliminary data. In this study, authentic X-ray radiographs of patients were obtained following ethical approval and subjected to preprocessing. The images were then annotated by orthopedic oncology specialists using advanced, state-of-the-art object detection algorithms trained with diverse architectural frameworks. All processes, from preprocessing to identifying pathological regions using object detection systems, underwent rigorous cross-validation and oversight by the research team. After performing various operations and procedural steps, including modifying deep learning architectures and optimizing hyperparameters, enchondroma formation in bone tissue was successfully identified. This achieved an average precision of 0.97 and an accuracy rate of 0.98, corroborated by medical professionals. A comprehensive study incorporating 1055 authentic patient data from multiple healthcare centers will be a pioneering investigation that introduces innovative approaches for delivering preliminary insights to specialists concerning bone radiography.

## Introduction

An enchondroma is a benign neoplasm of mature hyaline cartilage that extends from the medullary cavity to the cortical bone. This pathological formation can lead to significant endogenous mass formation in the bone tissue and is usually asymptomatic. However, enchondromas may present with different clinical symptoms depending on the size and location of the lesion and the features on radiological imaging. The detection and correct classification of enchondromas can significantly affect both the clinical practice and treatment of patients. Therefore, an accurate diagnosis of enchondromas is important for specialists, even though it is less dangerous than infections. Traditionally, enchondroma diagnosis has been based on clinical observations and various imaging techniques, particularly X-rays, computed tomography (CT), and magnetic resonance imaging (MRI). X-rays are commonly used to observe structural changes in bone tissue. X-ray imaging is recognized as the predominant and economically viable diagnostic modality among the various medical imaging techniques. It represents the most ubiquitous and readily available form of diagnostic imaging, which is economically advantageous and practical for medical practitioners, thereby establishing it as a fundamental component of medical imaging and a favored option for numerous healthcare professionals seeking effective diagnostic methodologies. The analysis and reporting of X-ray images conducted by radiologists and subsequently reviewed by specialized physicians generate a considerable workload, necessitating the involvement of multiple healthcare providers. However, detecting enchondroma-like lesions in X-ray images can be difficult, particularly for inexperienced observers. This can hinder timely diagnosis and prolong the treatment process. In this context, the use of advanced artificial intelligence (AI) and deep learning (DL) approaches promises to make the detection of such pathologies in X-ray images faster and more accurate^1^.

In recent years, deep learning techniques have transformed the field of medical image processing. These techniques have achieved high accuracy in object recognition, classification, and segmentation tasks by learning complex image patterns^2^. These technologies detect enchondromas, improve clinical accuracy, and provide healthcare professionals with valuable tools for interpreting images and managing treatment processes more effectively.

Although no existing studies have focused specifically on detecting enchondromas using current methodologies, the literature includes research on identifying various structures within bone tissue. These studies, which employed modern techniques, are summarized in Table 2 for comparative analysis and reference. The distinctions among these studies include variations in methodology, sample size, demographic focus, and the specific outcomes measured. These factors influence the overall findings and implications of each study. Yaholomi et al.^3^ trained a Faster-RCNN network for fracture detection using only 38 X-ray images and achieved an accuracy of 0.96 and an average precision of 0.866; however, the data were limited. He et al.^4^ showed that a large research group could use convolutional neural networks to discriminate three classes (benign-malignant- intermediate) of bone tumors on radiographs according to histopathological categories with an accuracy of 0.73. However, this study sorted the data according to different age groups, and the detection process consisted of 291 data points queried for different classes. Shukla and Patel^5^ used direct radiography and MRI in their study. Sobel, Canny, and Prewitt’s edge detection algorithms were used to visually detect the different types of cancer. They emphasized that tumor detection can be performed on direct radiographs after various applications of these methods, now called classical image-processing techniques, and that convolutional neural networks will play a more effective role in tumor detection in future studies. In a study by Chianca et al.^6^, primary bone tumors of the spine and spinal bone lesions in 146 patients were classified into three classes (benign, malignant, and primary malignant) using radiometric data mining on the Weka platform. The study compared deep artificial neural network classifications with the radiologist’s diagnosis and achieved success rates of 0.71 a 0.86 in the two classes. The study classified two classes with low classification success and did not provide information about the lesion in the other class, as in the previous examples. Anizusman et al.^7^ used 1200 images from 50 patients and classified three osteosarcoma tumors (necrotic, non-tumorous, and viable) with 90.3% success. Although the amount of data in the study seems to be sufficient, it is predicted that because of the limited number of patients from which the images were obtained, images with different characteristics belonging to the same patient were used. Therefore, due to data limitations, classifying data from 50 patients will not provide insight into real-world classification.

Sharma et al.^8^ used public X-ray image data (65 images with cancer and 45 healthy images, 105 images in total) and first identified the bone boundaries on the image using edge detectors, and then compared the cancer structure on detection using HOG and support vector machines. The study showed increased performance after support vector machines and HOG feature extraction, achieving 0.92 accuracy and 0.93 precision. This study was strengthened using cross-validation and different data-partitioning methods. However, this study differs from ours in that it used a small amount of visual data and did not include many deep learning methods. Cheng et al.^9^ aimed to detect bone metastases early using scintigraphy. A total of 576 images from cancer patients were used, and YOLO v4 was used. In the lesion-based evaluation, 0.90 precision and 0.72 sensitivity were obtained, while 0.94 precision and 0.92 sensitivity were obtained in the patient-based evaluation. Our study differs from ours in terms of the data type and scope. Scintigraphy images are more costly than X-ray images in terms of cost, accessibility, and limited data availability. The study’s data needs to be more comprehensive, and different deep learning methods have not been attempted.

Felfeliyan et al.^10^ performed bone and cartilage segmentation on knee MRI images of 500 patients using a Mask R-CNN. Success rates of 0.95–0.98 were obtained for bone segmentation and 0.71–0.80 for cartilage segmentation. Similar to our study of visual data and procedures, this study succeeded in segmenting different tissues. This differs from our study in terms of the data and detection points used. Gawade et al.^11^ used a convolutional neural network with VGG16-19, DenseNet201, and ResNet101 architectures to study 1144 osteosarcoma histological images from 50 patients. In this study, ResNet101 architecture was the most successful, achieving an accuracy metrics of 0.90 and a precision metric of 0.89. The accessible data consisted of ten-fold magnified microscopic images of pathologies from patients with tumors. This application, which is close to the scope of our study, provides visualization of the pathologies of patients with cancer detection and image classification. For this reason, the source data of the study is much more costly and requires different additional processing steps than our study in terms of cost and process. Xia et al.^12^ published their study on detecting cancer structure on the bone with mask R-CNN over 576 X-ray graphs, and 0.92 average accuracy was obtained in the segmentation study. The study did not apply the K-fold and was performed with limited data. Anttila et al.^13^ employed a deep-learning method to detect enchondroma structures in finger bones based on a dataset of 500 images. Despite the lack of information on the method and model details, the study reported that a detection rate of 0.90 was achieved, with an accuracy of 0.93.

This study examined the use of deep-learning techniques to detect enchondromas in X-ray images. For this purpose, a new approach to medical imaging using 1055 real patient data was presented. It provides valuable data to medical professionals for early diagnosis using radiographic images and improves clinical decision making. This approach also contributes to the ongoing digital transformation in healthcare by promoting the integration of deep learning techniques into medical diagnostic processes.

## Materials and methods

First, a statistical power analysis was performed to determine the sample size required to examine the detection of enchondromas using Deep Learning Approaches. G*Power software was used to perform the power analysis, and the results were determined at a confidence interval of 0.95 (p¡0.05). In addition, Cohen’s d-low effect size value was used as the basis for the paired t-test to be conducted in conjunction with the research^14^. The power values were calculated for various sample sizes, corresponding to an effect size of d = 0.200. Based on these values, a test power of approximately 0.994 was obtained with 500 observations. When the calculated power exceeded 0.80, this was statistically sufficient.

The images used in the study, were obtained from the archive of the Orthopedics and Traumatology Clinic at Ondokuz Mayıs University Hospital, were randomly selected and anonymized. The dataset consisted of the X-ray images of 1 055 different patients. Two orthopedic specialists labeled the area containing the enchondroma on the image. At this point, despite their limitations, radiologist reports were also taken into consideration. In total, 844 images in the dataset were randomly selected as the training dataset to train the model. The training dataset was validated using k-fold cross-validation (k = 5), with 844 training images and 211 validation images at each fold.

###  Pre-process

**Fig. 1:**
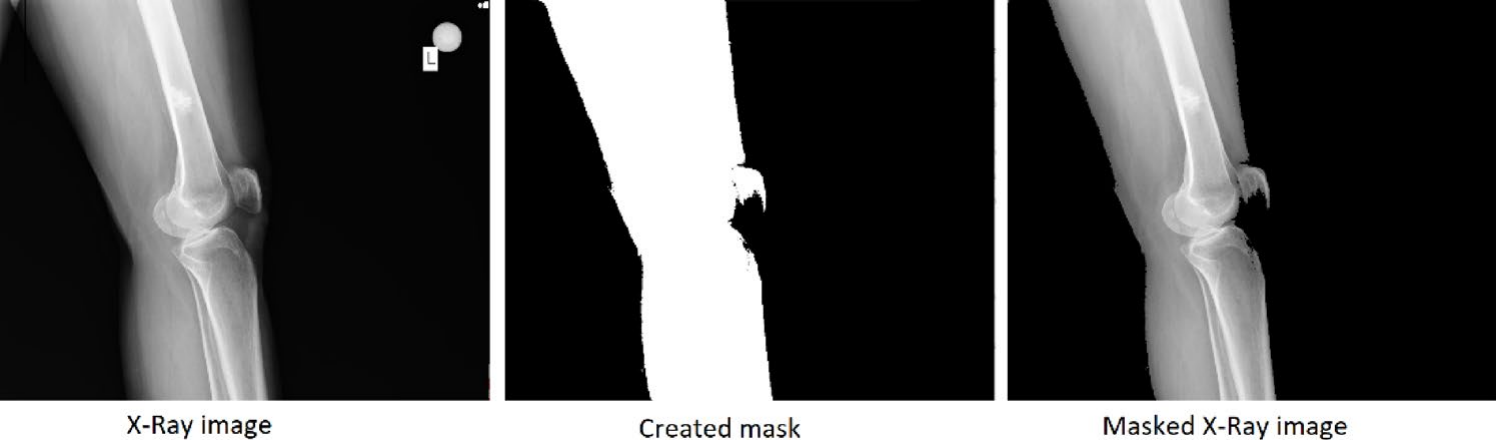
Image preprocess steps.

After collecting the data, they were anonymized and converted from a format containing various patient data to a format that contained only visual information. After these operations, the size and attributes of the data are reduced. However, performance and efficiency can be further improved by masking the area of interest. Figure 1 shows the visual and appropriate preprocessing steps.

The Canny and Prewitt edge filters were coded to mask only the area of interest by generating a binary mask in a high-resolution red, green, and blue (RGB) channel image. Masking the X-ray image improves image processing performance and removes some markings and defects from the image. After generating a binary mask for each image, the image was combined with the original RGB X-ray image and added to the dataset.

### The model

**Fig. 2:**
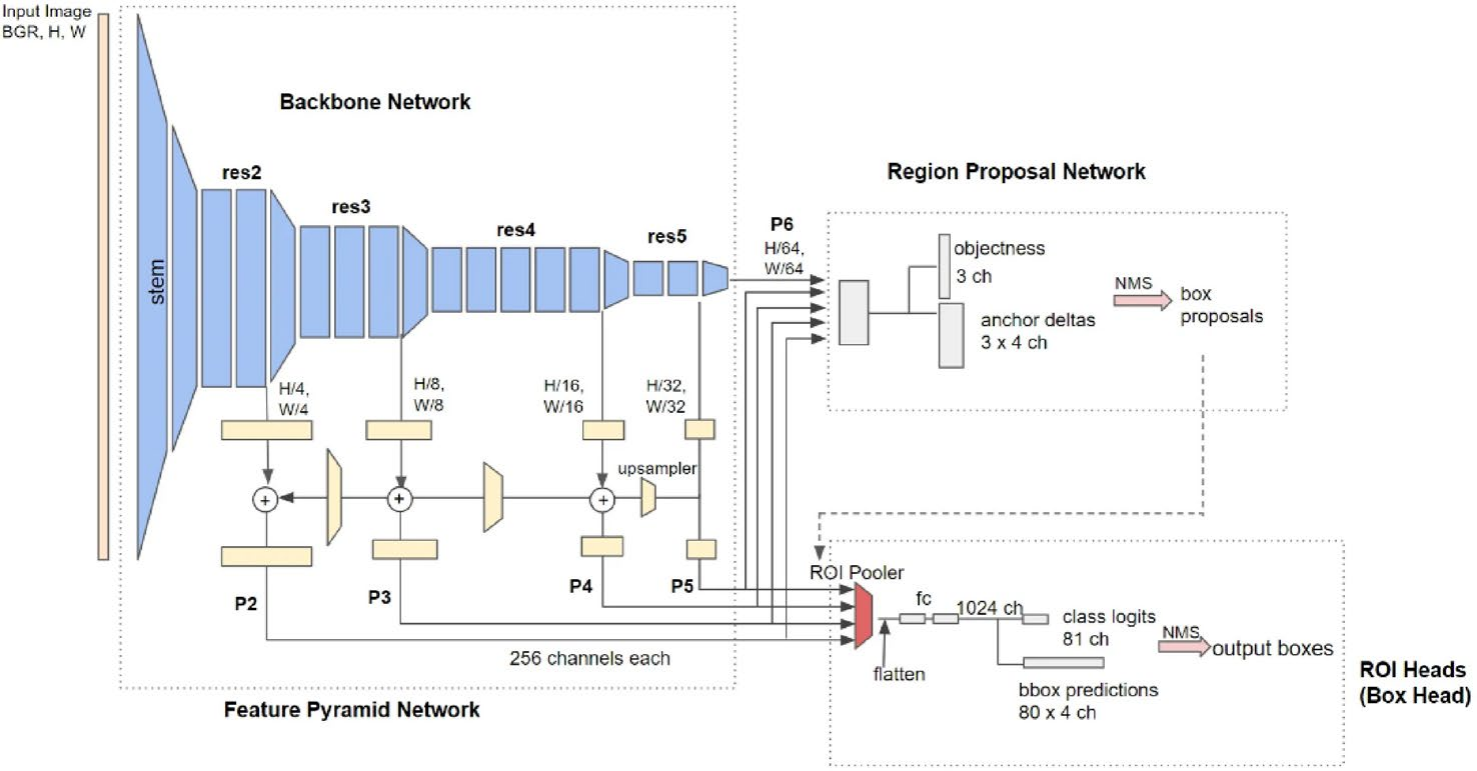
Diagram of the Detectron2 architecture. The backbone network provides feature maps (P1–P5) to the region proposal network (RPN). The ROI module identifies (bounding box) and separates (mask) the objects together with their respective classification^15^.

Detectron2 is a new-generation library of state-of-the-art detection and segmentation algorithms developed by Facebook AI Research. It is a successor to the Detectron and Mask R-CNN benchmarks. It can be employed in various research projects and production applications connected to computer vision, including Facebook. It incorporates the implementations of several object detection algorithms, including mask R-CNN, retinaNet, faster R-CNN, Region Proposal Network (RPN), Fast R-CNN, and TensorMask. The Detection 2 framework shown in Fig. 2 was analyzed across several segments. The Data Input Module is designed to load large amounts of data from the disk using optimization techniques such as caching and multi-worker systems. Users can also easily implement data augmentation techniques in the module’s data loader. In addition, the module is flexible, allowing users to customize and register their own datasets^15^. The backbone module obtains features from the provided images. This is accomplished using advanced convolutional neural networks, including ResNet or ResNeXt^16^. Customization of the module can enable the utilization of any standard convolutional neural network, which is effective for a specific image classification task. Notably, this module provides extensive insight into transfer learning. We could utilize pre-trained models in this context to employ a cutting-edge convolutional neural network that operates effectively with large image datasets such as ImageNet. Alternatively, we could use simpler networks in this module to improve efficiency regarding training and prediction times at the expense of accuracy.

The extracted features from the backbone are processed by Region Proposal, which proposes image regions with location specifications and scores. The scores indicate whether the regions contain objects with objectness scores. The objectness score of the proposed region can be either zero or one. The object score is specifically concerned with whether the region contains an object or background rather than its probability of being a class of interest^17^. We chose the YOLOv8 architecture for comparison based on the assumption that it would be the most successful for the task at hand. YOLOv8 is considered to be the most recent technological advancement. This is due to its higher mean average precision (mAP) and faster inference speed on the COCO dataset. An official paper has not yet been published.

It incorporates a newly developed neural-network architecture. It combines a Feature Pyramid Network (FPN) and a Path Aggregation Network (PAN). An additional annotation tool was introduced to streamline the annotation process and provide several valuable features, including auto-labelling, labeling shortcuts, and customizable hotkeys. Although the architecture is identical to that of its predecessor (version 6), many enhancements have been made in this version. These features were combined to create a more straightforward annotation of the images for training. Consequently, feature maps can be built with the ability to recognize objects at different scales and resolutions. A Feature Pyramid Network (FPN) progressively reduces the spatial resolution of the input image, while simultaneously increasing the number of feature channels^18^. In contrast, the PAN architecture uses skip connections to combine features from different levels of the network. This improves the network’s ability to capture features at different scales and resolutions, which is crucial for accurately detecting objects of different shapes and sizes.

## Results

###  Evaluation metrics

Among the various annotated datasets used in the detection of objects, the most common metric used by challenges and the scientific community to measure the accuracy of detection is the AP. There are different variants of AP. First, we explain the concept of AP^19^.

- True positive: Accurate identification of the ground truth bounding box was achieved.
- False positive: A non-existent object is incorrectly detected.
- False negative: An undetected object that should have been included in the ground-truth bounding box.

It is important to note that true negatives (TN) do not apply to object detection because images contain countless bounding boxes that need not be present. It is necessary to define ‘correct recognition’ and ‘incorrect recognition’ in the definitions presented. Intersection over union (IOU) is a standard method for evaluating the similarity coefficient of two datasets using the Jaccard index^20^. In object detection, the intersection over union (IOU) calculates the overlap between the predicted bounding box (Bp) and ground truth bounding box (Bgt). This was divided by union area.

1 $$J\left({B}_{p},{B}_{gt}\right)=IOU=\frac{area \left({B}_{p}\cap {B}_{gt}\right)}{area \left({B}_{p}\cup {B}_{gt} \right)}$$

Correct and incorrect detections can be determined by comparing their Intersection Over Union (IOU) with a predetermined threshold t. The detection is deemed correct if IOU is greater than or equal to t. If IOU is less than t, the detection is incorrect. It is worth noting that TNs are not utilized in object-detection frameworks. Consequently, metrics such as TPR, FPR, and ROC curves based on TNs should not be relied upon. The assessment of object detection techniques relies heavily on the principles of precision P and recall R, which are defined as follows.

2 $$P=\frac{TP}{TP+FP}=\frac{TP}{all \, detections}$$

3 $$R=\frac{TP}{TP+FN}=\frac{TP}{all \, ground \, truths}$$

Precision is the capacity of a model to exclusively recognize pertinent objects, as measured by the proportion of correct positive prophecies. Recall denotes the model’s ability to detect all applicable instances encompassing all ground-truth bounding boxes, measured by the proportion of correct positive predictions among all ground-truth cases. The precision-recall trade-off curve exhibits different confidence levels relating to the bounding boxes a detector generates in a graph where precision and recall are plotted. If the detector has low confidence, false positives are infrequent, resulting in an increased precision. However, this can result in many positives being missed. This leads to a high false-negative rate, and consequently, low recall. On the other hand, accepting more positives increases recall but also increases the false-positive rate and decreases precision. However, a good object detector should be able to find all ground-truth objects (FN = 0, high recall) while identifying only the relevant objects (FP = 0, high precision). This implies that the precision of the object detector must remain high when its recall increases. Hence, the precision and recall values should remain high even with varying confidence thresholds. As a result, a high area under the curve (AUC) generally indicates high precision and recall.^21^. In the definition of AP, instead of using the precision P(R) observed at recall level R for each different object identification, AP is obtained by considering the maximum precision, whose recall value is more significant than R. Moreover, interpolation can be performed over these points.

4 $${P}_{interp}(R)=\text{max}P ({R}^{\sim })$$

5 $$A{P}_{all}=\sum_{1}^{n}{({R}_{n+1}-{R}_{n})P}_{interp}({R}_{n+1})$$

The mean average precision (mAP) quantifies the accuracy of object detectors across all classes in a given database. This represents the mean accuracy (AP) of all classes^22,23^.

6 $$mAP=\frac{1}{n}\sum_{1}^{n}{AP}_{i}$$

### Model results and comparison

The data from 1055 patients were converted from DCM to PNG format, and ROIs were extracted to produce masked images. The images were resized to a standard 1024 × 1024 pixel size. Table 1 displays the YOLO and DETR2 parameters used for training. Several different parameters were tested to obtain the specified parameters. Finally, the hyperparameters listed as success and performance were obtained. ROI extraction and masking led to improved performance and success in YOLO. However, this did not significantly increase the detector success rate. Nevertheless, it yielded a measurable reduction in the processing time when tested in experiments with 2000 or more epochs. Using the parameters in Table 1, YOLO v8 requires a learning process of 85 min, whereas Detectron2 takes 64 min with a Tesla K80 24 GB GPU.

**Table 1:** Train parameters.

Maximum iteration = 2000
Evaluation period = 200
Base Learning rate = 0.001
Roi heads batch size per image = 64
Data loader worker = 2
Training example per iteration = 2

**Fig. 3:**
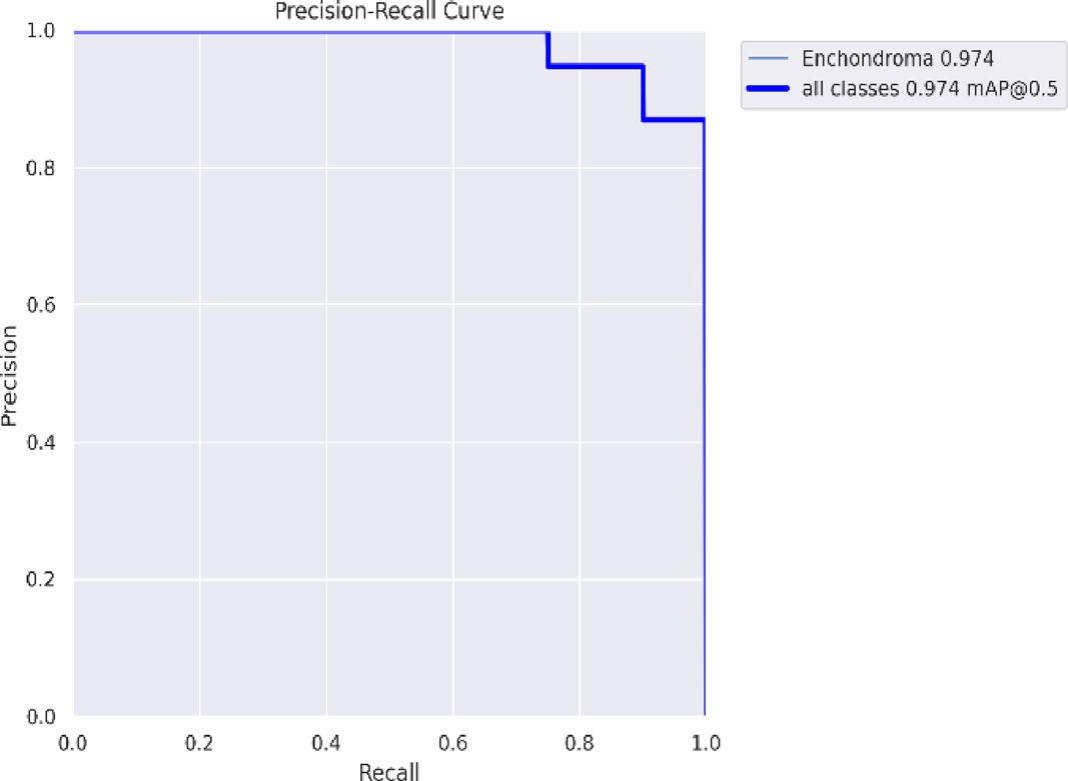
YOLO v8 Model result PR curve.

**Fig. 4:**
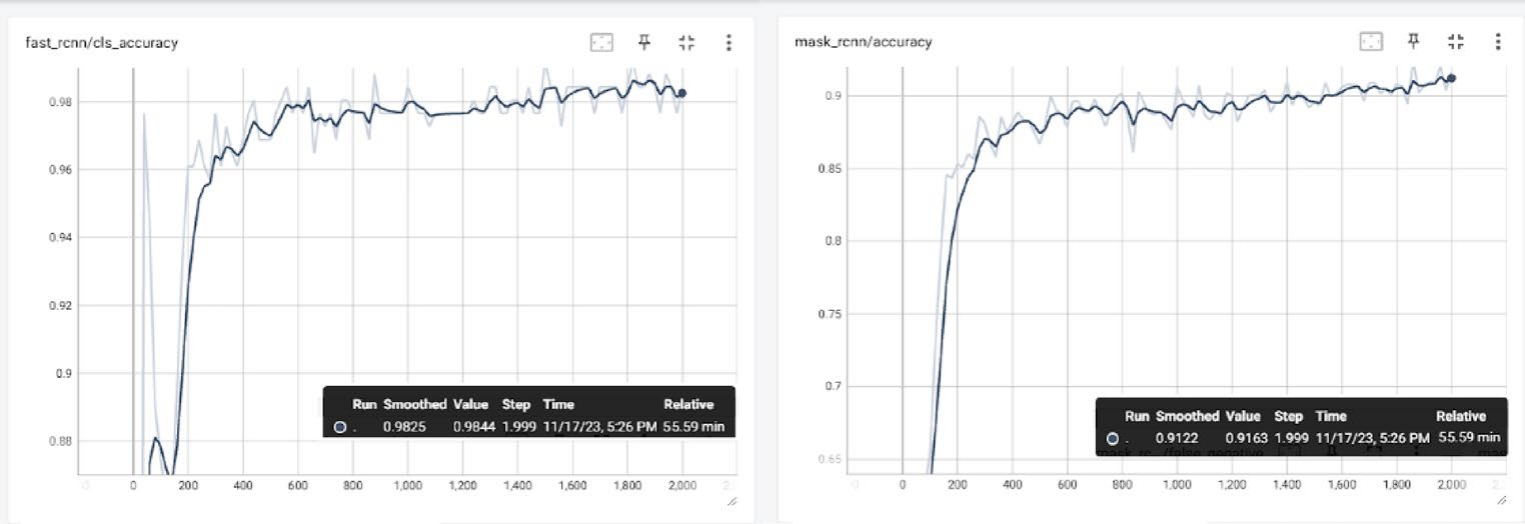
Detectron accuracy curves.

**Fig. 5:**
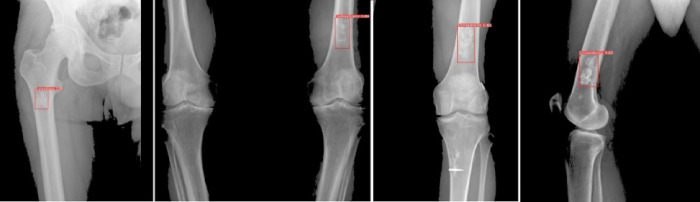
YOLO v8 prediction samples.

The metrics shown in Figs. 3 and 5 were obtained by evaluating Detectron2 and YOLO v8. In YOLO v8, the prediction labels are given in a square shape, whereas in Detectron, which runs Mask R-CNN and Faster R-CNN, the enchondroma area is colored and marked in a square shape. The method, which offers the superiority of Mask R-CNN in object detection and reporting, has provided the opportunity to achieve higher success with its accuracy value. The PR curve of YOLOv8 is shown in Fig. 3, with an average precision of > 0.97. Figure 4 also shows the Fast R-CNN, Mask R-CNN, and accuracy curves, showing the accuracy values obtained with Detectron2.

Figure 3 shows the PR slope of YOLO v8 with an average precision of > 0.97. In YOLO v8, the prediction labels are given as squares and the detected enchondroma structure remains within this label. A success rate of more than 95% proves that this method provides sufficient support to decision-making experts and sufficient success at the reporting point. In contrast, in Detectron, which runs Mask R-CNN and Faster R-CNN, the enchondroma area is colored and marked as a square. After parameter optimization, the performance value increased to above 0.98, proving that the system is much more stable and successful. The robustness of the results is reinforced by the results obtained after the K-fold cross-validation. Figure 4 also shows the accuracy curves, which show the accuracy values obtained using Fast R-CNN, Mask R-CNN, and Detectron2. In addition to the precision and accuracy values obtained on a metric basis, the detection and inference of images in the test data were also significant, as discussed in the Discussion section.

## Discussion

Orthopedic surgeons commonly evaluate patients with undetermined pain due to intraosseous cartilage tumors; however, missing malignancy is possible during the examination. Enchondroma is the second most common benign bone tumor, accounting for 0.3 of bone tumors and 0.13 of benign bone tumors^24^. Although the age distribution is 5–80, most cases are between the third and fifth decades, and both genders are equally affected^25^. Cartilage tumors are usually asymptomatic and form hyaline cartilage. They are primarily located in the metaphysis and diaphysis and rarely in the epiphysis of the short and long tubular bones of the limbs^26^. They typically present as solitary lesions and are often discovered incidentally during radiographic examinations for unrelated reasons. Olier disease and Maffucci syndrome are associated with multiple enchondromas^25^. They are often detected incidentally on radiography, magnetic resonance imaging (MRI), or scintigraphy. Lesions on the hands and feet cause symptoms such as painful or painless soft tissue swelling and pathological fractures, whereas lesions on the long bones are mostly asymptomatic^27^. Radiography of the two planes should always be the first imaging method. When diagnosis is difficult, MRI and CT should be used; calcification, periosteal bone formation, cortical destruction, and soft tissue can be better visualized^28^.

Enchondromas exhibit variable appearances on imaging. Extended endosteal scalloping, cortical destruction, pathological fracture, and periosteal reaction are suspicious evidence of a malignant tumor on standard X-rays. On CT scans, characteristic features include lytic areas, cortical lesions with scalloping greater than two-thirds of the cortex, and soft tissue extension. MRI can evaluate the medullary spread of the tumor and detect periosteal reactions and surrounding edema^29^. In the differential diagnosis, bone infarction, chondrosarcoma, benign lytic bone lesions, lytic metastasis, and granulomatous disease should be considered^27^. Most enchondromas remain asymptomatic and do not require further treatment. If cortical thinning is detected during follow-up or at first diagnosis, surgery should be performed without waiting for a fracture. Aggressive treatment is used in patients with malignant transformation^30^. Most patients with malignant transformation complained of pain; however, some did not. This knowledge of the malignant transformation of enchondromas indicates that identifying and treating this tumor is critical^28^.

Depending on the patient’s complaints, X-rays are one of the first diagnostic tools for orthopedic cases^31^. Even in the absence of symptoms, detecting bone tissue structures is valuable for follow-up and prevention. However, the density of radiological reporting systems has increased exponentially with the advent of Covid-19. This increase has led to different outcomes, such as interpreting radiographs in different centers, presenting to specialists and patients, and lengthening the reporting processes^32^. Using a machine learning method trained on big data from real patients to support reporting systems or providing radiologists with prior knowledge through a subroutine will help reduce workload and prevent potential human errors.

**Fig. 6:**
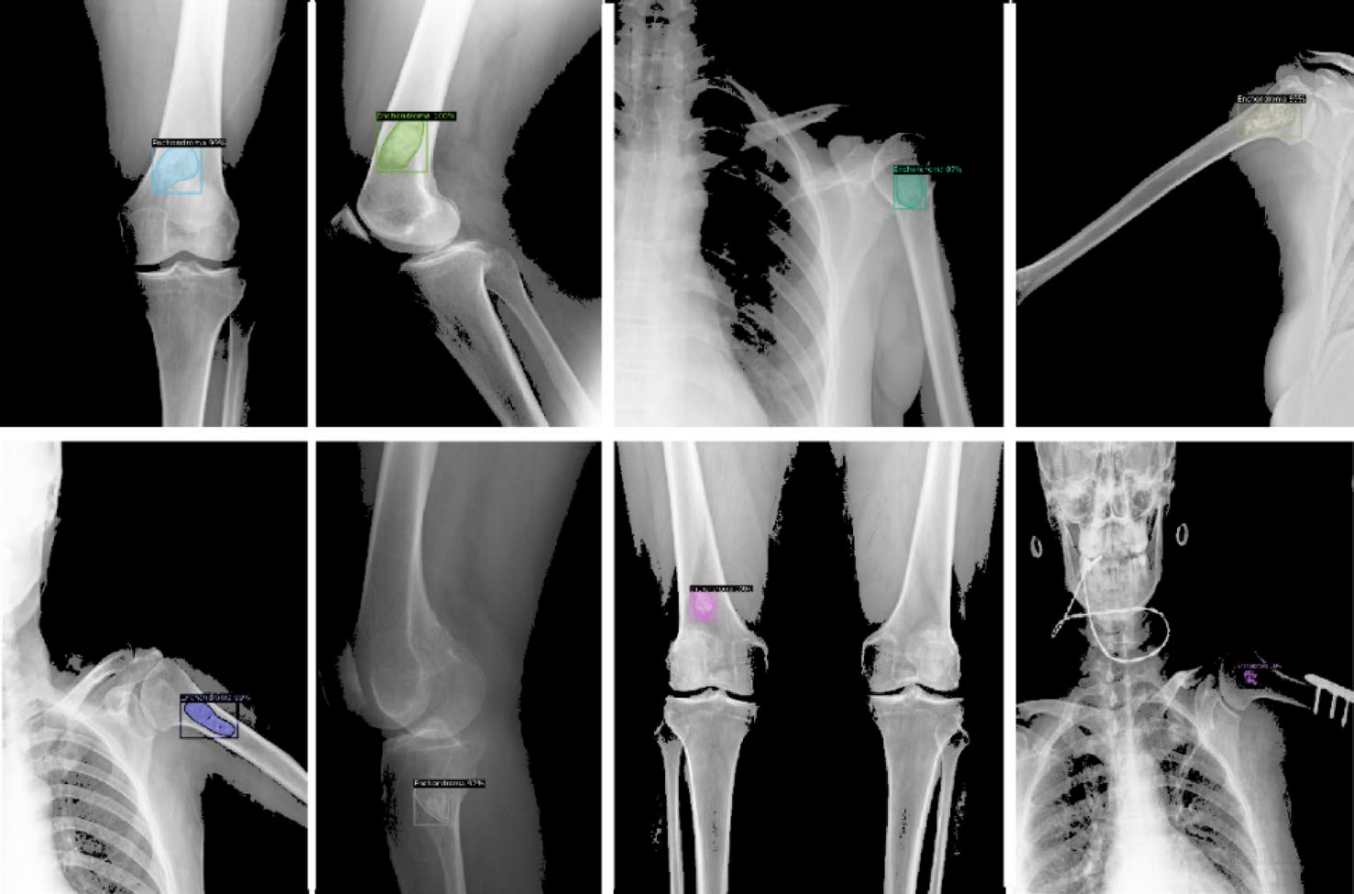
Detectron prediction samples.

One of the reasons for detecting nonsymptomatic structures in our study was that additional information and follow-up data were obtained from radiographs performed by experts for different diagnoses. Figure 5 shows a few X-rays of the inference results obtained by training the YOLO v8 model. Although it is less accurate than Detectron, it can still serve as a decision-support system. Figure 6 shows examples of the inference results of the Detectron 2 model.

Detection and segmentation with high accuracy, even in radiographs containing different anomalies, has been highly successful. With over 0.98 accuracy, it is the preferred decision support and reporting model. The deep learning model achieved an accuracy of 0.98 with faster training processes than various abnormality detection methods in the literature. With these characteristics and using the deep learning model, our study differs from the studies presented in Table 2 and offers superior success.

**Table 2:** Deep learning-based object detection in medical radiology.

References	Year	Aim	Dataset	Architecture	Evaluation
Yaholomi et al.^3^	2019	Fracture detection	38	Faster R-CNN	0.96 Accuracy, 0.866 mAP
He et al.^4^	2020	Bone tumor classification	2899	EfficientNet	0.746 Accuracy
Shukla and Patel^5^	2020	Bone cancer detection	16	Edge detection	Preliminary study, no evaluation
Do et al.^33^	2021	Bone tumor detection	1061	Multi-Level Seg-Unet	0.84 IoU
Eweje et al.^34^	2021	Bone lesions classification	1060	EfficientNet	0.76 accuracy, 0.81 sensitivity, 0.90 specificity
Chianca et al.^6^	2021	Bone tumors classification	146	Weka platform	0.80 Sensitivity, 0.75 Specificity
Anisuzzaman et al.^7^	2021	Bone tumor classification	1200	VGG16-19	0.93 Accuracy
Sharma et al.^8^	2021	Bone tumors classification	105	Edge detection + SVM	0.92 Accuracy
Cheng et al.^9^	2021	Bone metastases diagnosis	576	Yolo4	0.90 Accuracy, 0.92 Precision
Felfeliyan et al.^10^	2022	Bone osteoarthritis segmentation	500	Mask R-CNN	0.90 mAP
Gawade et al.^11^	2023	Bone cancer detection	1144	ResNet101	0.90 Accuracy, 0.89 Precision
Xia et al.^12^	2023	Bone cancer detection	576	Mask R-CNN	0.92 mAP
Anttila et al.^13^	2023	Enchondroma detection	500	Not specified	0.93 Accuracy
Sampath et al.^35^	2024	Bone tumor detection	1141	AlexNet	0.98 Precision
Hong et al.^36^	2024	Bone tumor detection	112	Adaptive Boosting	0.90 Precision
Gassert et al.^37^	2025	Bone tumor classification	344	R-CNN	0.75 Accuracy

In their study, Hong et al. used a very limited number of CT-based images to differentiate between enchondromas and atypical cartilaginous tumors. CT and MRI are advanced methods in the detection of cartilaginous tumors^36^. Our X-ray-based dataset, which includes lesions in long and flat bones, is one of the strengths of our study. Thanks to its highly reliable lesion detection, it promises a cost-effective result. In the literature, Gaspert et al. also focused on classification in their study using a limited amount of CT-based data^37^. To classify chondroid tumors, studies with larger datasets are needed, if possible, to analyze MRI and CT images together.

This study has some limitations. In our study, X-ray images of patients with a radiological or pathological diagnosis of enchondroma were included. In the future, deep learning methods could be used to analyze data from patients with chondroid matrix tumors at different stages, as well as those with progression in long-term follow-up, to obtain information on the differential diagnosis and follow-up of these tumors. The strengths of our study are its methodology, and the use of a large dataset compared to literature. Additionally, the study included pathologies in different locations, not only in long bones but also in bones such as the skull and ribs.

In conclusion, this study achieved superior detection accuracy (0.9899) compared to previous literature. Enchondroma follow-up can therefore be used to assess the risk of recurrence and malignant transformation in growing lesions. However, the use of advanced imaging methods may be limited in the evaluation of clinically and radiologically stable lesions.

## Data Availability

The data used in this study were collected and processed by the researchers and were approved by Ondokuz Mayis University Clinical Research Ethics Committee (Ref Code: OMU KAEK 2022/120). For verification and permission requests, please contact Ayhan AYDIN (https://github.com/AyhanAydinPhD). According to the Regulation on the Protection of Personal Health Data, researchers are prohibited from disclosing their data to third parties. It can be used for scientific studies with permission from relevant institutions. The authors shared the codes produced in this study following the project completion. This can be requested through the Ayhan AYDIN’s GitHub page or e-mail.

## References

[1] Ballabriga, R. et al. Photon counting detectors for x-ray imaging with emphasis on ct. *IEEE Trans. Radiat. Plasma Med. Sci.* **5**, 422–440 (2020).

[2] Maurya, S. et al. A review on recent developments in cancer detection using machine learning and deep learning models. *Biomed. Signal Process. Control* **80**, 104398 (2023).

[3] Yahalomi, E., Chernofsky, M. & Werman, M. Arai, K., Bhatia, R. & Kapoor, S. (eds) Detection of distal radius fractures trained by a small set of x-ray images and faster r-cnn. (eds Arai, K., Bhatia, R. & Kapoor, S.) *Intelligent Computing* (2019).

[4] He, Y. et al. Deep learning-based classification of primary bone tumors on radiographs: A preliminary study. *EBioMedicine* **62**, 103121 (2020). 33232868 10.1016/j.ebiom.2020.103121 PMC7689511

[5] Shukla, A. & Patel, A. Bone cancer detection from x-ray and MRI images through image segmentation techniques. *Int. J. Recent Technol. Eng.* **8**, 273–278 (2020).

[6] Chianca, V. et al. Radiomic machine learning classifiers in spine bone tumors: A multi-software, multi-scanner study. *Eur. J. Radiol.* **137**, 109586 (2021). 33610852 10.1016/j.ejrad.2021.109586

[7] Anisuzzaman, D., Barzekar, H., Tong, L., Luo, J. & Yu, Z. A deep learning study on osteosarcoma detection from histological images. *Biomed. Signal Process. Control* **69**, 102931 (2021).

[8] Sharma, A. et al. Bone cancer detection using feature extraction based machine learning model. *Comput. Math. Methods Med.* **2021**, 74 (2021). 10.1155/2021/7433186 PMC8712164 34966444

[9] Cheng, D.-C., Hsieh, T.-C., Yen, K.-Y. & Kao, C.-H. Lesion-based bone metasta- sis detection in chest bone scintigraphy images of prostate cancer patients using pre-train, negative mining, and deep learning. *Diagnostics* **11**. https://www.mdpi.com/2075-4418/11/3/518 (2021). 10.3390/diagnostics11030518 PMC8000593 33803921

[10] Felfeliyan, B., Hareendranathan, A., Kuntze, G., Jaremko, J. L. & Ronsky, J. L. Improved-mask R-CNN: Towards an accurate generic MSK MRI instance segmentation platform (data from the osteoarthritis initiative). *Comput. Med. Imaging Graph.* **97**, 102056 (2022). 35364383 10.1016/j.compmedimag.2022.102056

[11] Gawade, S., Bhansali, A., Patil, K. & Shaikh, D. Application of the convolutional neural networks and supervised deep-learning methods for osteosarcoma bone cancer detection. *Healthc. Analyt.* **3**, 100153 (2023).

[12] Xia, G., Ran, T., Wu, H., Wang, M. & Pan, J. The development of mask r-cnn to detect osteosarcoma and oste-ochondroma in x-ray radiographs. *Comput. Methods Biomech. Biomed. Eng: Imaging Vis.* **11**, 1–7 (2023).

[13] Anttila, T. T. et al. Enchondroma detection from hand radiographs with an inter- active deep learning segmentation tool—A feasibility study. *J. Clin. Med.* **12**, 7129 (2023). 38002741 10.3390/jcm12227129 PMC10672653

[14] Faul, F., Erdfelder, E., Lang, A.-G. & Buchner, A. G* power 3: A flexible statistical power analysis program for the social, behavioral, and biomedical sciences. *Behav. Res. Methods* **39**, 175–191 (2007). 17695343 10.3758/bf03193146

[15] Wu, Y., Kirillov, A., Massa, F., Lo, W.-Y. & Girshick, R. Detectron2. https://github.com/facebookresearch/detectron2 (2019).

[16] Khan, A., Sohail, A., Zahoora, U. & Qureshi, A. S. A survey of the recent architectures of deep convolutional neural networks. *Artif. Intell. Rev.* **53**, 5455–5516 (2020).

[17] Shangguan, Z. & Rostami, M. Improved region proposal network for enhanced few-shot object detection. arXiv preprint arXiv:2308.07535 (2023). 10.1016/j.neunet.2024.106699 39243514

[18] Reis, D., Kupec, J., Hong, J. & Daoudi, A. Real-time flying object detection with yolov8. arXiv preprint arXiv:2305.09972 (2023).

[19] Padilla, R., Netto, S. L. & Da Silva, E. A. A survey on performance metrics for object-detection algorithms, 237–242 (IEEE, 2020).

[20] Jaccard, P. E´tude comparative de la distribution florale dans une portion des alpes et des jura. *Bull. Soc. Vaudoise Sci. Nat.* **37**, 547–579 (1901).

[21] Lin, T.-Y., Goyal, P., Girshick, R., He, K. & Doll´ar, P. Focal loss for dense object detection, 2980–2988 (2017). 10.1109/TPAMI.2018.2858826 30040631

[22] Ren, S., He, K., Girshick, R. & Sun, J. Faster R-CNN: Towards real-time object detection with region proposal networks. Advances in Neural Information Processing Systems **28** (2015). 10.1109/TPAMI.2016.2577031 27295650

[23] Liu, W. et al. *SSD: Single Shot Multibox Detector* 21–37 (Springer, 2016).

[24] Cao, X., Ren, Q., Li, X., Tian, Y. & Wang, Z. Epiphyseal enchondroma masking as osteoid osteoma: A case report. *Eur. J. Med. Res.* **26**, 1–5 (2021). 33962677 10.1186/s40001-021-00504-y PMC8106184

[25] O’Connor, M. I. & Bancroft, L. W. Benign and malignant cartilage tumors of the hand. *Hand Clin.* **20**, 317–323 (2004). 15275690 10.1016/j.hcl.2004.03.019

[26] Douis, H. & Saifuddin, A. The imaging of cartilaginous bone tumours. I Benign lesions. *Skelet. Radiol.* **41**, 1195–1212 (2012). 10.1007/s00256-012-1427-0 22707094

[27] Omlor, G. W. et al. Outcome of conservative and surgical treatment of enchondromas and atypical cartilaginous tumors of the long bones: Retrospective analysis of 228 patients. *BMC Musculoskelet. Disord.* **20**, 1–12 (2019). 30922289 10.1186/s12891-019-2502-7 PMC6440168

[28] Herget, G. et al. Insights into enchondroma, enchondromatosis and the risk of secondary chondrosarcoma. Review of the literature with an emphasis on the clinical behaviour, radiology, malignant transformation and the follow up. *Neoplasma* **61**, 365–378 (2014). 24645839 10.4149/neo_2014_046

[29] Parlier-Cuau, C., Bousson, V., Ogilvie, C. M., Lackman, R. D. & Laredo, J.-D. When should we biopsy a solitary central cartilaginous tumor of long bones? literature review and management proposal. *Eur. J. Radiol.* **77**, 6–12 (2011). 21241899 10.1016/j.ejrad.2010.06.051

[30] Tang, C., Chan, M., Fok, M. & Fung, B. Current management of hand enchondroma: A review. *Hand Surg.* **20**, 191–195 (2015). 25609298 10.1142/S0218810415300028

[31] Pfeifer, R. & Pape, H.-C. Missed injuries in trauma patients: A literature review. *Patient Saf. Surg.* **2**, 1–6 (2008). 18721480 10.1186/1754-9493-2-20 PMC2553050

[32] Grasselli, G., Pesenti, A. & Cecconi, M. Critical care utilization for the covid-19 outbreak in lombardy, Italy: early experience and forecast during an emergency response. *JAMA* **323**, 1545–1546 (2020). 32167538 10.1001/jama.2020.4031

[33] Do, N.-T., Jung, S.-T., Yang, H.-J. & Kim, S.-H. Multi-level seg-unet model with global and patch-based x-ray images for knee bone tumor detection. *Diagnostics* **11**, 691 (2021). 33924426 10.3390/diagnostics11040691 PMC8070216

[34] Eweje, F. R. et al. Deep learning for classification of bone lesions on routine MRI. *EBioMedicine* **68**, 103402 (2021). 34098339 10.1016/j.ebiom.2021.103402 PMC8190437

[35] Sampath, K., Rajagopal, S. & Chintanpalli, A. A comparative analysis of CNN-based deep learning architectures for early diagnosis of bone cancer using CT images. *Sci. Rep.* **14**(1), 2144 (2024). 38273131 10.1038/s41598-024-52719-8 PMC10811327

[36] Hong, R., Li, Q., Ma, J., Lu, C. & Zhong, Z. Computed tomography-based radiomics machine learning models for differentiating enchondroma and atypical cartilaginous tumor in long bones. In *RöFo-Fortschritte auf dem Gebiet der Röntgenstrahlen und der bildgebenden Verfahren* Vol. 186 (Georg Thieme Verlag KG, 2024).

[37] Gassert, F. G. et al. A deep learning model for classification of chondroid tumors on CT images. *BMC Cancer* **25**(1), 561 (2025). 40155859 10.1186/s12885-025-13951-1 PMC11951610

